# Subjective and Objective Evaluation of Surface Properties of Flattened Bamboo and Polyurethane Self-Foaming Plastic

**DOI:** 10.3390/polym17070894

**Published:** 2025-03-26

**Authors:** Yushu Chen, Qianwei Liang, Jinjing Wang, Xinyu Ma

**Affiliations:** College of Furnishings and Industrial Design, Nanjing Forestry University, Nanjing 210037, China; 13450161671@163.com (Q.L.); 13952572696@163.com (J.W.); 13338613620@163.com (X.M.)

**Keywords:** bamboo instead of plastic, flattened bamboo, PU self-foaming plastic, surface characteristics, perceptual evaluation

## Abstract

With the increasing demand for environmental protection, flattened bamboo is gradually attracting attention as a sustainable material. The purpose of this study was to compare and analyze the surface properties of flattened bamboo and PU self-foaming plastic by subjective and objective evaluation methods, and to explore the substitutability of flattened bamboo and PU self-foaming plastic in furniture design. Objective test methods such as surface hardness testing, gloss measurement, and friction coefficient determination were used in the experiments, and the subjective evaluation of visual and tactile perception of the materials were combined with the semantic differential method. It was found that the flattened bamboo was generally superior to the PU self-foaming plastics in terms of color, gloss, roughness, and wettability, giving a more delicate, warm and comfortable feeling, while the PU self-foaming plastics stood out in terms of personalized style. Further correlation analysis showed that surface gloss and color saturation had a significant effect on the users’ psychological feelings. This study provides a scientific basis for replacing PU self-foaming plastic with bamboo in furniture design and also provides a valuable reference for “bamboo instead of plastic” product design.

## 1. Introduction

PU (polyurethane) self-foaming plastic is a type of semi-rigid foam, which is widely used in furniture manufacturing. It plays an important role in the production of public chairs and bar seat backrests [1,2]. The advantages of PU self-foaming plastic are its simple process, high production efficiency, and a certain softness and elasticity, but its main disadvantages are environmental pollution and that it is difficult to recycle [3]. The production of PU self-foaming plastic relies on chemical synthesis, and the foam structure has a high internal surface area, which makes it prone to fire at high temperatures, and it is non-recyclable, resulting in environmental pollution [4].

In contrast, bamboo, as a natural renewable material with biodegradability, good mechanical and processing properties, and unique texture aesthetics [5], is available in a variety of forms such as round bamboo, bamboo slices, gabions, flattened bamboo, bamboo bundles, bamboo filaments, bamboo pulp fibers, and so on [6], and therefore shows a broad application prospect in furniture design [7]. In recent years, with the enhancement of environmental awareness and the deepening of the concept of sustainable development, China’s National Development and Reform Commission and other departments have issued a three-year action plan to accelerate the development of “Replacing plastic with Bamboo”, aiming to apply the outstanding advantages and role of bamboo in replacing plastic products and reducing plastic pollution. “Bamboo instead of plastic” [8] has gradually become an important direction to promote sustainable product design [9]. As a form of bamboo material, flattened bamboo has the characteristics of easy processing and superior mechanical properties. For furniture design, bamboo and PU self-foaming plastic have certain commonality in the processing form, and show a complementary relationship in performance and application, which provides the possibility of using flattened bamboo as a substitute for PU self-foaming plastic to achieve better safety and sustainability. In addition, from an economic point of view, bamboo, as a fast-growing renewable resource, has relatively low planting and processing costs. Although the processing cost of rapidly prototyping PU self-foaming plastics is low, the cost will increase due to the impact of density and quality. With the continuous development and promotion of “bamboo instead of plastic” technology, the production cost of “bamboo instead of plastic” furniture is expected to be further reduced.

However, there are differences between flattened bamboo and PU self-foaming plastic in terms of the surface properties and feelings during usage, so it is important to analyze their surface properties in detail and in a comparative manner. Current research has mostly focused on people’s visual perception of furniture surface materials [10,11,12,13,14], and less research has been conducted on the connection between them and surface properties. Through experimental data and subjective evaluation, this study provides an in-depth discussion on the substitutability of flattened bamboo and PU self-foaming plastic in furniture design. Clarifying the performance and advantages of “bamboo instead of plastic” materials help to develop more bamboo-based innovative products, meet the market demand for environmentally friendly products, provide a scientific basis for future bamboo furniture design, and promote the transformation of the industry towards a green and sustainable direction.

## 2. Materials and Methods

### 2.1. Sample Preparation

In this study, PU self-foaming plastic and flattened bamboo were selected as the research objects. PU self-foaming plastic was supplied by Foshan Oshujian Furniture Manufacturing Co., Ltd., Foshan, Guangdong, China. The sample dimensions were 100 mm (length) × 80 mm (width) × 12 mm (thickness). The density of the samples was about 380–450 kg/m^3^, and the dimensions were 100 mm (length) × 80 mm (width) × 12 mm (thickness).

Flattened bamboo was provided by Yu Shu wooden Crafts Factory, Heze, China, with the specifications of 100 mm (length) × 80 mm (width) × 10 mm (thickness), and the flattened bamboo substrate was sanded with 500 mesh sandpaper and dusted with a wool brush, coated with primer and varnish (primer for 2 times, topcoat for 1 time, and sanded again for 1 time after each coat was dried), and dried at room temperature for 8 h. The eight samples used in the experiment are named in Table 1.

### 2.2. Experimental Apparatus

- Colorimeter: SEGT-J portable colorimeter (Tianchuang Instrument Co., Ltd. from Zhuhai, China);
- Gloss meter: HG268 gloss meter (3nh Technology Co., Ltd. from Shenzhen, China);
- Roughness meter: TR100 pocket surface roughness meter (Horiyang Precision Measurement Co., Ltd. from Shanghai, China);
- Contact Angle Measuring Instrument (Zhongchen Digital Technology Equipment Co., Ltd. from Shanghai, China).

### 2.3. Experimental Methods

#### 2.3.1. Color Test

A SEGT-J colorimeter was used to measure the color of the samples and record the Lab values. Since PU self-foaming plastic samples come in a variety of colors, four colors, grey, orange, green, and blue, which are used more frequently in practice, were selected for this test. Three points were taken on the surface of each sample, and the average value was measured and recorded. The measurement and calculation of the material color parameters used the CIE (1976) L*a*b* color system [15]. The higher the value of L*, the higher the brightness of the object. For completely white objects, the L value is 100, and for completely black objects, the L value is 0. For the red and green color index a*, a larger positive value indicates that the color is more red, while a larger negative value indicates that the color is more green. For the yellow and blue color index b*, a larger positive value indicates that the color is more yellow, while a larger negative value indicates that the color is more blue [16,17]. By referring to the method of Sadoku et al. to convert the data into Menzel color space values [18], the conversion method was as follows:

(1) H=−0.03636L*+0.02663r−14.3θ+0.09131rθ+14.826

(2) V=0.1002L*−1.16

(3) $$C=0.1439r+1.054\theta -1.022\theta^{2}+0.497r\theta -0.167$$

(4) $$\theta =\arctan\left(\frac{a*}{b*}\right)$$

(5) $$r=\sqrt{a{*}^{2}+b{*}^{2}}$$

where V is the brightness value, H is the hue scale value, C is the color saturation, and r and θ are intermediate variables used in the conversion. H is a quantitative scale value based on YR, and when the value is in the range from 0 to 10, the hue can be expressed as HYR (e.g., 5.6 YR); when −10 < H < 0, the hue scale is (H + 10)R; when 10 < H < 0, the color phase is (H + 10)R; when 10 < H < 20, the color phase is (H − 10)Y; when 20 < H, the color phase is (H − 10)GY.

#### 2.3.2. Glossiness Test

Glossiness was tested according to ISO (2014) using the HG268 gloss meter [19]. The optical geometric condition selected for the measurement was a 60° angle of incidence, and the direction of incidence of the light source was selected to be parallel to the grain (GZL) and perpendicular to the grain (GZT) [20]. Three points were taken on the surface of each specimen, these values were recorded, and the difference between gloss values parallel to the texture direction and gloss values perpendicular to the texture direction was compared.

#### 2.3.3. Roughness Test

The surface roughness of the specimens was tested using a pocket surface roughness tester TR100, and the sampling length was set to 0.8 mm [21]. In order to improve the accuracy, three points were selected on each specimen for testing, the arithmetic mean deviation (Ra) value of each specimen contour was read, and the average value was taken as the roughness test result of the specimen.

#### 2.3.4. Surface Wettability Testing

The results of the surface wettability test were expressed using the contact angle. The contact angle is the angle between the tangent line of the droplet at the edge of solid-liquid contact and the solid plane. The size of the contact angle value can be used to measure the hydrophilicity and hydrophobicity of the solid surface, generally 90° as the demarcation line; when 0° < Ө < 90°, the solid surface shows hydrophilicity. The greater Ө is, the greater the hydrophobicity is, i.e., the better the water resistance.

When testing the surface wettability, the specimen was first placed on the carrier table in the longitudinal direction, the angle of the specimen was adjusted so that it was perpendicular to the water droplet syringe, and about 2 μL of the liquid to be tested was taken from the sampling control system of the contact angle meter and dropped on the surface of the specimen; the liquid used was the homemade distilled water in the laboratory. The software ADVANCE (https://www.kruss-scientific.com/en/products-services/advance-software, accessed on 16 February 2025) was employed to record the shape of the liquid droplet at the instant of its contact with the sample. Subsequently, the measurement tool within the software was utilized to measure the arc of the liquid droplet [22]. Three different positions on the sample surface were randomly selected for testing and the average value was taken as the test result.

#### 2.3.5. Subjective Evaluation Test

The subjective evaluation test was based on the Semantic Difference (SD) method, which aims to understand people’s intuitive feelings and real experiences of the material through different perceptual words [23,24]. The specific steps in this method were as follows:

1. Perceptual language pair establishment

Through the literature, websites, and other channels, we collected several words about the users’ impressions of furniture materials, then referred to the experts’ opinions to eliminate synonyms, screened out the perceptual imagery words that best represented subjects’ subjective impressions of the furniture materials, and added antonyms to form perceptual pairs to form a semantic difference scale [25].

2. Perceptual semantic evaluation

Using a questionnaire in the form of a seven-level scale, subjects were invited to observe and touch the PU self-foaming plastic and flattened bamboo samples, and were asked to give each sample a subjective score from −3 to 3 points according to their subjective perceptual impression. If the score was closer to the two extreme values, it meant that the subject’s perception of the material was stronger, and if the score was at “0”, it meant that the user’s evaluation of the material was neutral.

## 3. Results

### 3.1. Surface Performance Results Analysis

#### 3.1.1. Color Test Results

A material’s color is a natural property that reflects the visual characteristics of its surface and causes psychological feelings [26]. The results of the sample color test are shown in Table 2. The L* value of the flattened bamboo was between 60 and 70, while that of PU self-foaming plastic was between 40 and 70, and both materials possessed high brightness. When selecting colors for actual furniture, especially public furniture, attention will be paid to the enhancement of color for design recognition, and the use of bright and eye-catching colors can strengthen the visual recognition of furniture in public environments [27,28].

According to Menzel’s color system, the color tone value of the flattened bamboo was in the YR range and the value was between five and ten, which belonged to the yellow-red system and had a warm tone; among the tested PU self-foaming plastics, the samples of B1 and B2 were in the YR range and the value was low, which indicated that the color had a blue-greenish cold tone; B3 was in the YR range and the value was high, which had a warm tone. B4 was in the R range of the red system, and the value was low, which had a greenish cold tone. Through comparison, it was seen that the color tone of the flattened bamboo was warm, while the color tone of the PU self-foaming plastic was more variable, with both cold and warm tones.

In terms of color saturation, the C-value of the flattened bamboo was between 12 and 14, showing moderate saturation, while the C-value of PU self-foaming plastic had a wider range, from 1.69 to 35.10, showing differences in color saturation. Overall, the color saturation of the flattened bamboo was moderate, while the saturation of the PU self-foaming plastic varied greatly depending on the color of the samples.

#### 3.1.2. Gloss Test Results

**Figure 1:**
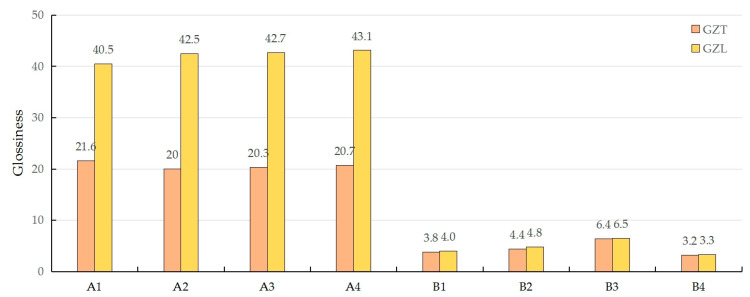
Comparison of the parallel and perpendicular texture gloss of the specimens.

The data in Figure 1 show that the gloss of the flattened bamboo was significantly higher than that of the PU self-foaming plastic, and in the test direction, the vertical texture gloss of the PU self-foaming plastic and the parallel texture gloss were not significantly different, while the parallel gloss of bamboo was slightly higher than the vertical gloss. This was because the water-based film-forming paint material on the surface of the bamboo greatly enhanced the glossy feeling of the material. When the light rays are incident perpendicularly along the texture, the light that enters the cell lumen is reflected and then blocked by the cell wall, resulting in a greater degree of scattering of the reflected light, which results in a smaller amount of gloss on the surface of the bamboo; when the light rays are incident parallel to the texture of the bamboo, a part of the light will be refracted from the cells along the direction of the long axis of the cells, and then the light is blocked by the cell wall. Due to refraction, the possibility of light being blocked by the cell wall is smaller than when it is perpendicular to the grain, which improves the reflectivity of light and makes the glossiness higher [29]. The epidermal layer of the PU self-foaming plastic has a rough texture, which makes the light scatter many times on the surface to form a diffuse reflection, which reduces the glossiness.

#### 3.1.3. Roughness Test Results

**Figure 2:**
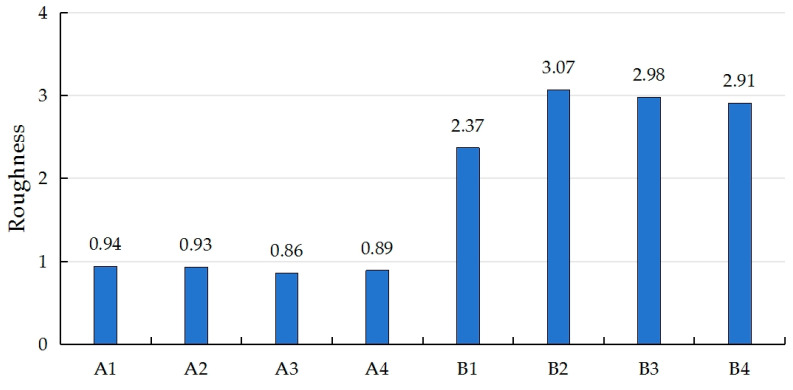
Comparison of the specimens’ roughness.

As shown in Figure 2, the surface roughness of the flattened bamboo was significantly lower than that of the PU self-foaming plastic, which was due to the fact that the flattened bamboo is sanded and painted before use, which makes the surface flatter and smoother and greatly reduces the roughness. The chemical substances in the PU self-foaming plastic, such as polyurethane raw materials and foaming agents, will form specific chemical structures during the mixing and reaction process, and these structures further evolve into the surface texture during foaming, thus forming a surface effect with a certain roughness [30].

#### 3.1.4. Surface Wettability Test Results

**Figure 3:**
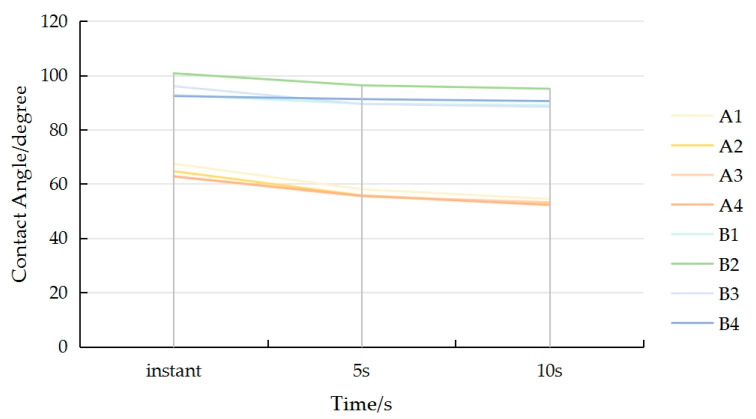
Trend for the contact angle of the samples.

**Figure 4:**
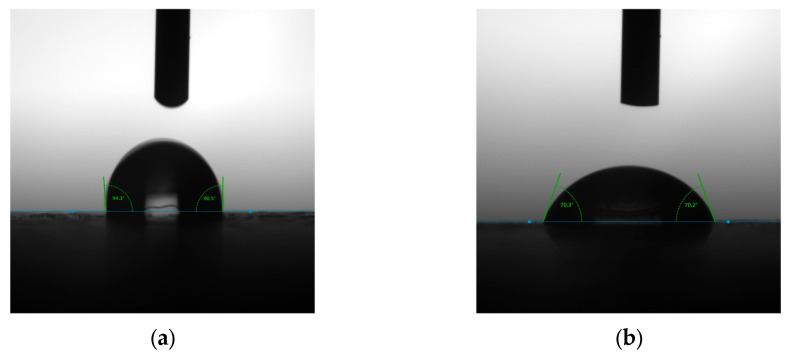
Schematic diagram of the samples’ contact angle. (**a**) Contact Angle Diagram of PU self-foaming plastic, (**b**) Contact Angle Diagram of flattened bamboo.

It can be seen from the test data shown in Figure 3 and Table 3, the contact angle of the PU self-foaming plastic was above 90°, and the contact angle changed less with the duration of time, which shows hydrophobicity. The surface contact angle of the flattened bamboo was smaller than that of the PU self-foaming plastic, which was lower than 90° and continued to spread over 10 s, showing hydrophilicity and stronger surface wettability. This is because the molecular chain of polyurethane materials contain certain hydrophobic groups, such as ester groups, which are enriched on the surface and can reduce the polarity of the surface, thus improving the hydrophobicity; the pigment contained in the colorful polyurethane is also hydrophobic, and the increase in filler content will make the contact angle increase [31]. Bamboo is affected by its own chemical composition, porous structure and other characteristics, and the material itself has strong hygroscopicity and water absorption, so the contact angle would be relatively small [32].The differences in the images of the contact angles can be seen in Figure 4.

### 3.2. Analysis of the Subjective Evaluation Results

#### 3.2.1. Perceptual Vocabulary Screening and Categorization

In order to better allow users to make a perceptual evaluation of furniture materials, based on the review of the materials science literature, the search of design psychology websites, and questionnaire research, we collected 50 words about the imagery of furniture surface materials, invited a number of furniture designers and scholars specializing in furniture design to conduct interviews and discussions, and formulated a number of principles for the selection of the perceptual vocabulary: (1) The selected words can reflect the visual and tactile characteristics of a certain feeling, each representing a certain psychological feeling; (2) The selected words can correspond more comprehensively to the visual and tactile parameters of the furniture surface materials; (3) The selected words are closely related to the role of furniture in meeting people’s spiritual needs.

According to the above criteria, after screening and removing the words with high semantic repetition or low relevance, six groups of representative perceptual pairs were finally identified and divided into sensory and psychological dimensions, as shown in Table 4.

#### 3.2.2. Subjective Evaluation Test Results

The semantic differential method was used to design the questionnaire [33], and the questionnaire form was a seven-level scale, which required subjects to rate the degree of lexical perception of the six groups of words for the surface of each sample according to their own subjective impressions; the greater the score, the stronger the intensity of the subject’s perception of a certain right-side perceptual word corresponding to the sample, and vice versa, the weaker the score, the stronger the degree of perception of a certain left-side perceptual word corresponding to the sample.

**Figure 5:**
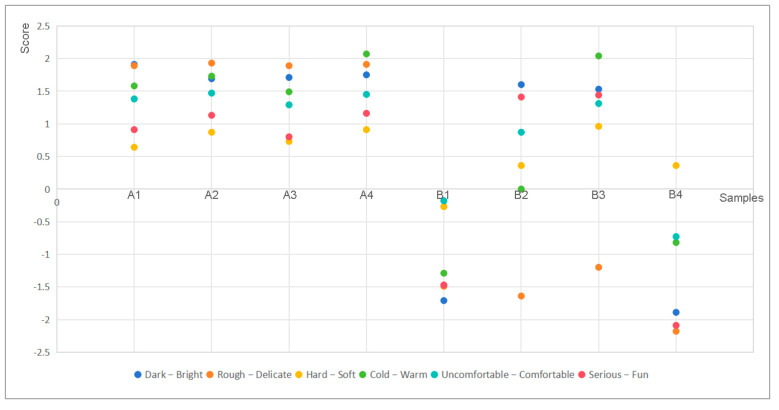
Scatter plot of the samples’ evaluated mean scores.

Subjects were assigned perceptual semantic evaluations of the samples at the same location. A total of forty-seven sets of samples were collected, among which two invalid questionnaires were excluded, and finally the number of valid questionnaires was forty-five. In terms of the gender of the subjects, there were 18 males and 27 females aged between 18 and 30, and the background structure of the subjects was reasonable. Finally, the 45 pieces of data collected by the questionnaire were sorted out and imported into the software Excel 2013 to calculate the mean values of the six groups of perceptual imagery vocabulary representing the samples (Table 5), and to construct a scatter plot of the samples’ evaluation means, with the x-axis as the number of the sample, and the y-axis as the evaluation mean value of each perceptual imagery vocabulary pair in the corresponding samples, as shown in Figure 5.

The data were imported into SPSS 2.0 software for reliability analysis, which showed that the Cronbach alpha coefficient was 0.921, which was greater than 0.9, indicating that the data in this study had a high degree of reliability and could be used for further analysis.

The results of the analysis in Figure 5 show that people feel differently about the two materials. For the options of “Rough–Delicate” and “Dark–Bright”, people’s ratings for the flattened bamboo were significantly higher than those for the PU self-foaming plastic, which was consistent with the results of the roughness and gloss tests, i.e., the flattened bamboo with low roughness gives a more delicate feeling, while the higher gloss brings a brighter visual effect. In the “Hard–Soft” option, the highest rating was given to the B3 PU self-foaming plastic sample, even though the flattened bamboo was rated relatively high. All in all, the flattened bamboo was able to bring a better surface feeling in terms of sensory experience. For the options of “Warm–Cold” and “Uncomfortable–Comfortable”, flattened bamboo was rated higher than the majority of the PU self-foaming plastics. In the “Warm–Cold” and “Uncomfortable–Comfortable” options, flattened bamboo scored higher than most of the PU self-foaming plastic, but the B3 PU self-foaming plastic sample still received a higher score, demonstrating an outstanding sense of warmth and comfort. For the option of “Serious–Fun”, the ratings of the PU self-foaming plastics samples B2 and B3 were significantly higher than the ratings of the other samples, while the ratings of B1 and B4 were lower, showing greater differences compared with B2 and B3; and, the ratings of the flattened bamboo were in the range of 0.8–1.2. This suggested that different PU self-foaming plastics can present strong individualized style attributes, while the flattened bamboo shows a balanced and moderate style effect. To summarize, the flattened bamboo can bring a better sensory experience and more positive psychological feelings, but there is still room for improvement in the overall effect, especially in the style and emotional expression of the material; and, the connection between the material properties and user perception should be further explored in order to obtain more detailed data.

### 3.3. Correlation Analysis Between Surface Properties and Subjective Evaluation

In order to analyze the relationship between objective surface performance and subjective evaluation, the correlation coefficients between various physical attribute parameters and the semantic scores of subjective adjectives were calculated, which helped to reveal whether there was a correlation between them [34]. The physical parameters of the samples in the objective performance test and the semantic scores of the six subjective perceptual adjectives were selected as the variables and imported into the SPSS software for Pearson correlation analysis to obtain the data in Table 6.

As an analytical method widely applied in the field of statistics, Pearson correlation analysis aims to characterize the strength of the mutual relationship between different variables. In this analysis, it can intuitively reflect, in terms of numerical values, the relationship between the surface properties of the two materials and the subjective evaluation. From the results of the correlation analysis, it was seen that the physical characteristics of the material had a significant effect on the psychological perception. Glossiness had the most significant correlation with “Dark–Bright”, where increasing glossiness enhanced the feeling of brightness, and had an effect on the perception of warmth, comfort and delicacy. Roughness had a strong negative correlation with “Rough–Delicate” and no significant effect on the rest of the perceptions, i.e., roughness did not significantly contribute to the overall perception of the material. Among the chromaticity values, the brightness L* value showed some significance and positive correlation with a number of subjective ratings and a high brightness improved the overall evaluation, while the a* value of red tones mainly affected people’s perception of “warmth”, and the b* value of yellowish-blue tones had a greater impact on the feelings of softness, finesse, warmth, and comfort. This indicated that the color of the material is an important factor influencing people’s subjective feelings, which also explains why the B3 samples with a higher saturation and warm tones had higher scores in the psychological dimension. In addition, the correlation between the contact angle and the “Cold–Warm” and “Rough–Delicate” options were also high and negative. A smaller contact angle represents higher surface wettability and brings a warm and delicate touch, so the hydrophilic flattened bamboo had a softer and wetter surface touch, and thus had higher scores.

**Figure 6:**
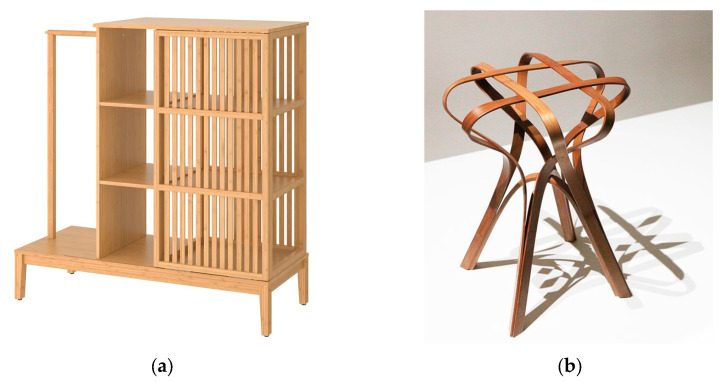
Different styles of bamboo furniture. (**a**) Original style furniture with low gloss and light colors, (**b**) Mild style furniture with high gloss and dark colors.

The analysis results in Figure 5 and Table 6 show that, in the comparison of the two materials, the flattened bamboo was outstanding in the sensory scores, but slightly ordinary in the psychological scores, and was especially lacking in the senses of fun and warmth. Therefore, in the material treatment and product design of “bamboo instead of plastic”, it is necessary to consider the breakthrough of style to achieve richer psychological feelings on the basis of good sensory evaluation. In the present results, material color and glossiness showed high correlation with several subjective evaluation indexes, and related studies also show that people perceive these two factors faster than other factors [35]. Therefore, for the manufacturer, according to the grasp of the design style, the focus should be placed on the gloss and color to improve the effect. In terms of gloss, the gloss level of bamboo can be controlled by grinding, painting with wooden wax oil to present different visual effects. Similarly, in terms of color, manufacturers can try to change the surface color of bamboo by carbonization, bleaching, or dyeing to adjust the brightness and tone of the bamboo color [36]. For example, as shown in Figure 6, for a rough and natural styles, a lower gloss could be used and the primary color of the bamboo could be maintained, retaining the original texture; for gentle, delicate, and refined styles, higher gloss and darker colors could be used to add atmosphere and sophistication to the product. In addition, manufacturers can also show a more diversified style by matching with other materials. For example, choosing metal materials with strong color contrast with bamboo can not only highlight the color effect of the bamboo, but also create a modern texture. Fabrics of the same color system can also be added to enrich the color level while highlighting the overall sense of interest or warmth.

## 4. Discussion

This study focused on the comparative analysis of the objective performance and subjective evaluation of flattened bamboo and PU self-foaming plastics to find out the differences between the two and to seek ways to improve the evaluation of flattened bamboo, so as to promote the development of “bamboo instead of plastic” in furniture design. The results of this study are summarized and discussed within the following three aspects:

1. Objective performance comparison

Through experimental tests and comparisons, it was seen that the flattened bamboo was superior to the PU self-foaming plastics in both gloss and roughness, i.e., it had higher gloss and lower roughness, presenting a better visual effect and surface tactile sensation. In the surface wettability test, the wettability of the flattened bamboo was high, while its hydrophobicity was slightly lower than that of the PU self-foaming plastics, showing a certain degree of hydrophilicity. Therefore, in the subsequent “bamboo instead of plastic” material treatment, it was considered to improve the sealing property of the material by improving the surface process, such as applying protective wax, paint, etc. One can also reduce the hydrophilic groups inside the bamboo by drying treatment, or hot steam treatment, to increase the density and hardness of the bamboo, thereby enhancing the hydrophobicity of the material. In the color test, the flattened bamboo had high brightness and saturation, and had an overall warm tone, while the PU self-foaming plastics, according to the different colors, had obvious differences in brightness, hue, and saturation; as such, the specific color feeling needed to be analyzed in conjunction with the subjective evaluation. On the whole, in the surface performance of the material, flattened bamboo was slightly better than the PU self-foaming plastics and had better development prospects in the “bamboo instead of plastic” product design.

2. Subjective evaluation analysis

It was seen from the subjective evaluation results that the flattened bamboo had a prominent score in the “Dark–Bright” and “Rough–Delicate” options, and a higher score in the “Hard–Soft”, “Cold–Warm”, and “Uncomfortable–Comfortable” options, and a lower score in the “Serious–Fun ” option. The overall scores for the PU self-foaming plastics were all low, but samples B2 and B3 scored significantly higher in “Serious–Fun ”. In general, the score for the flattened bamboo was high in the sensory dimension, but low in the psychological dimension. This is because flattened bamboo as a natural material, although having good surface properties, is limited to a certain extent in the color performance; as the color adjustment range of the PU self-foaming plastics is larger, it can more fully show the interest and other different style characteristics.

3. Correlation analysis between objective performance and subjective evaluation

Through the correlation analysis of the objective performance and subjective evaluation, it was seen that the physical characteristics measured in the experiment had a certain impact on psychological feelings. The physical characteristics that had the most obvious impact on each feeling were mainly concentrated in gloss and chroma values, and were positively correlated with the subjective scores, while the roughness and contact angle had little impact on the scores and were negatively correlated. Therefore, in the product design of “bamboo instead of plastic”, the focus of the material treatment can be placed on gloss and color to shape a more diverse product style. In glossiness, the gloss level of the flattened bamboo is controlled to present different visual effects. In terms of color, one can adjust the brightness and tone of the flattened bamboo or combine it with other materials to change the overall color effect.

4. Research limitations

There were some potential limitations in this study, and the collected data can be affected by experimental conditions, researchers, and survey methods. If it is necessary to reduce the bias and make the experimental results more comprehensive and accurate, diversified methods can be adopted in subsequent studies. For example, these techniques can be combined with the corresponding physiological data measurement methods, such as EEG experiments. Through EEG experiments, the changes in the electrical activity of the brain can be monitored in real time when the user is in contact with the “bamboo instead of plastic” furniture, so as to accurately capture the user’s subconscious reaction to different material textures and designs. This data based on human physiological response can provide a deeper and objective basis for the optimization of furniture design. In addition, it can also show the effect of materials for furniture through three-dimensional rendering, and show the appearance, texture, and other characteristics of the materials in actual furniture applications by simulating the effect of different lighting and viewing angles, so as to enrich the evaluation content and further provide more valuable design and production support for the “bamboo instead of plastic” furniture market.

For consumers, the surface properties of the materials have a significant impact on the perception and evaluation of furniture. Therefore, for the use of “bamboo instead of plastic” furniture surface materials, it is necessary to jointly consider the material properties and user experience of both materials. By measuring the objective physical properties and subjective psychological evaluations of the flattened bamboo and the PU self-foaming plastics and conducting correlation analysis, the intrinsic relationships between the surface gloss, color, roughness, and surface wettability of the materials and the subjective evaluations were obtained. On this basis, it is proposed that in the design of “bamboo instead of plastic” furniture, it is necessary to enhance the hydrophobicity of the material and consider improvements in the surface effect in terms of glossiness and color, so as to improve the competitiveness of bamboo furniture and promote the development and application of environmental protection and consciousness in furniture design.

## Figures and Tables

**Table 1:** Sample naming and number.

Serial Number	Sample	Name
1	Flattened bamboo 1	A1
2	Flattened bamboo 2	A2
3	Flattened bamboo 3	A3
4	Flattened bamboo 4	A4
5	PU self-foaming plastic 1	B1
6	PU self-foaming plastic 2	B2
7	PU self-foaming plastic 3	B3
8	PU self-foaming plastic 4	B4

**Table 2:** Color test data.

Samples	L*	a*	b*	H	V	C
A1	63.7	14.5	33.1	8.93 YR	5.22	12.71
A2	66.57	14.2	33	8.89 YR	5.51	12.51
A3	63.9	15.0	32.0	8.60 YR	5.24	12.87
A4	62.8	15.15	32.3	8.65 YR	5.13	13.01
B1	42.1	2.2	2.2	2.37 YR	3.06	1.69
B2	68.3	−19.1	−14.0	1.59 YR	5.68	14.37
B3	56.4	47.7	45.0	7.75 YR	4.49	35.10
B4	44.8	−9.03	−0.8	4.53 R	3.33	7.14

**Table 3:** Sample contact angle data.

Time	A1	A2	A3	A4	B1	B2	B3	B4
instant	67.45	64.67	62.67	62.81	92.80	100.77	96.0	92.39
5 s	58.04	55.77	55.38	55.63	89.53	96.34	89.45	91.25
10 s	54.44	52.64	53.23	52.20	89.02	95.08	88.4	90.52

**Table 4:** Subjective evaluation of the perceptual language pairs.

Serial Number	Representative Perceptual Pairs	Evaluation Dimension
1	Dark–Bright	Sensory
2	Rough–Delicate	Sensory
3	Hard–Soft	Sensory
4	Cold–Warm	Psychological
5	Uncomfortable–Comfortable	Psychological
6	Serious–Fun	Psychological

**Table 5:** Evaluated mean scores for the perceptual language comparison.

Representative Perceptual Pairs	Score							
	A1	A2	A3	A4	B1	B2	B3	B4
Dark–Bright	1.91	1.69	1.71	1.75	−1.71	1.6	1.53	−1.89
Rough–Delicate	1.89	1.93	1.89	1.91	−1.49	−1.64	−1.2	−2.18
Hard–Soft	0.64	0.87	0.73	0.91	−0.27	0.36	0.96	0.36
Cold–Warm	1.58	1.73	1.49	2.07	−1.29	0	2.04	−0.82
Uncomfortable–Comfortable	1.38	1.47	1.29	1.45	−0.18	0.87	1.31	−0.73
Serious–Fun	0.91	1.13	0.8	1.16	−1.47	1.41	1.44	−2.09

**Table 6:** Correlation analysis of objective physical parameters and subjective semantic scores.

	GZT	GZL	Roughness	L*	a*	b*	Contact Angle
Dark–Bright	0.682 *	0.647 *	−0.502	0.937 ***	0.411	0.581	−0.505
Rough–Delicate	0.995 ***	0.994 ***	−0.974 ***	0.634 *	0.344	0.681 *	−0.968 ***
Hard–Soft	0.618	0.597	−0.43	0.658 *	0.619	0.77 **	−0.527
Cold–Warm	0.754 **	0.72 **	−0.59	0.709 **	0.716 **	0.873 ***	−0.637 *
Uncomfortable–Comfortable	0.747 **	0.714 **	−0.601	0.856 ***	0.553	0.714 **	−0.593
Serious–Fun	0.527	0.491	−0.342	0.897 ***	0.431	0.521	−0.336

Ps: ***, **, * represent 1%, 5%, and 10% significance levels, respectively.

## Data Availability

The raw data presented in this study are available on request from the author.

## References

[1] Ates M., Karadag S., Eker A.A. (2022). Polyurethane foam materials and their industrial applications. Polym. Int..

[2] Fu X.Y., Huang Z.Y., Lv Q.Z., Wang Y.H. (2024). Sustainable Design Strategies of Educational Furniture Based on LCA Methodology. Packag. Eng..

[3] Pau D.S.W., Fleischmann C.M., Delichatsios M.A. (2020). Apparatus for investigating the burning and dripping of vertically oriented polyurethane foams. Fire Mater..

[4] Wang X.Z., Lu M.S., Zeng J.B., Weng Y.X., Li Y.D. (2021). Malleable and thermally recyclable polyurethane foam. Green Chem..

[5] Hakeem K.R., Ibrahim S., Ibrahim F.H., Tombuloglu H. (2015). Bamboo biomass: Various studies and potential applications for value-added products. Agricultural Biomass Based Potential Materials.

[6] Hu Y.A., Huang H., He L., Pan C.Y., He M. (2024). Research status and development trends of “bamboo as as ubstitute for plastic” in construction and building materials. J. For. Eng..

[7] Zheng Y., Zhu J. (2021). The application of bamboo weaving in modern furniture. BioResources.

[8] National Development and Reform Commission, Ministry of Industry and Information Technology, Ministry of Finance, State Forestry and Grass Administration, etc (2023). Accelerate the Development of “Bamboo Instead of Plastic” Three-Year Action Plan. https://www.gov.cn/zhengce/zhengceku/202311/content_6913316.htm.

[9] Ye H.Z., Fu J., Cheng H.T., Chen F.M., Yang S.Y. (2024). The Current Status and Market Development Potential of Processing Technology and Products Using Bamboo as a Substitute for Plastic. Sci. Silvae Sin..

[10] Wan Q., Hu Q., Chen B., Fang H., Ke Q., Song S. (2021). Study on the visual cognition of laminated bamboo furniture. For. Prod. J..

[11] Tang T., Fei B., Song W., Su N., Sun F. (2022). Tung Oil Thermal Treatment Improves the Visual Effects of Moso Bamboo Materials. Polymers.

[12] Zhang M., Yuan X.F. (2024). Material Perception Experience in Furniture Product Design: A Literature Review. Furnit. Inter. Des..

[13] Xue G., Guo Z., Xie Z. (2022). Using Kansei Engineering for the Design Thinking Framework: Bamboo Pen Holder Product Design. Sustainability.

[14] Yang Y.N., Li J., Guo M.Y., Wei X., Huang Z.L., Liu Y., Guo H.W. (2023). Quantitative Evaluation of Visual Perception Semantic Fractal Dimension of Wood Materials. Packag. Eng..

[15] Su N., Fang C., Zhou H., Tang T., Zhang S., Wang X., Fei B. (2021). Effect of Rosin Modification on the Visual Characteristics of Round Bamboo Culm. Polymers.

[16] Liu R., Zeng Z., Li Q., Hu C. (2022). The effects of ACQ and water glass on the color change and decay resistance of carbonized bamboo. Wood Res..

[17] Yan X., Chang Y., Qian X. (2020). Effect of Concentration of Thermochromic Ink on Performance of Waterborne Finish Films for the Surface of Cunninghamia Lanceolata. Polymers.

[18] Ken S. (1985). Conversion from Measured Values by Machine to Visual Values: Transformation from the L*, a*, b* Color System to Munsell. Wood Ind..

[19] (2014). Paints and Varnishes—Determination of Gloss Value at 20 Degrees, 60 Degrees and 85 Degrees, CEN/TC 139—Paints and Varnishes.

[20] Lin X.Y., Zhang Q.H., Huang Y.H., Huang Q.F., Yang X. (2022). Painting technology and adhesion mechanism of waterborne paint based on bamboo laminated lumber. J. Beijing For. Univ..

[21] Dagher R., Stevanovic T., Landry V. (2023). Wood color modification with iron salts aqueous solutions: Effect on wood grain contrast and surface roughness. Holzforschung.

[22] Jiang J.C., Li J., Zhang J.Y., Wang B.Q., Zhan J.F. (2024). Effect of Oil Wax Heat Treatment on Physical Properties and Dimensional Stability of Wood. For. Eng..

[23] Zhang Y., Guo Y., Wei P., He Z., Yi S., Zhao G. (2024). Effect of changes in surface visual properties of heat-treated wood on the psychological preference. BioResources.

[24] Nagai T., Matsushima T., Koida K., Tani Y., Kitazaki M., Nakauchi S. (2015). Temporal properties of material categorization and material rating: Visual vs non-visual material features. Vis. Res..

[25] Zhou X., Bai R., Jin Y., Xiao W., Xue C. (2023). Aesthetic Cognitive Computing Clues of Materials Based on Multidimensional Perception. J. Test. Eval..

[26] Lian C., Wang X., Chen H., Fei B., Pang X., Lian J., Wu Z. (2022). Using Statistical Methods to Comparatively Analyze the Visual Characteristics of Flattened Bamboo Boards in Different Bamboo Culms. Polymers.

[27] Leung M.Y., Famakin I.O., Wang C. (2019). Developing an integrated indoor built environment-quality of life model for the elderly in public and subsidized housing. Eng. Constr. Archit. Manag..

[28] Ahmed H.T., Aly A.M. (2023). Recycled Waste Materials in Landscape Design for Sustainable Development (Al-Ahsa as a Model). Sustainability.

[29] Yu X.N., Liu S., Chen B.X., Bao X.D., Guan X., Lin J.G. (2024). Study on Effect of Sanding Treatment on Surface Properties of Zenia insigins Wood. China For. Prod. Ind..

[30] Lan P., Qiao L.L., Liao A.P., Nong Y., Qin R.D., Liu Q., Li H.B., Chen H. (2015). Effect of lanthanum ricinoleate on the performance of water-blownintegral skin polyurethane foams. Chem. Ind. Eng. Prog..

[31] Członka S., Sienkiewicz N., Kairytė A., Vaitkus S. (2019). Colored polyurethane foams with enhanced mechanical and thermal properties. Polym. Test..

[32] Lu S.W., Jian Y.L., San H., Liu Y.G., Chai X.J., Xu K.M., Xie L.K. (2022). Silylation of Moso Bamboo(Phyllostachys Edulis) Surface and Preventable Wettability and Penetration for Water and Oil. Surf. Technol..

[33] Ding M., Cheng Y., Huang X.G., Zhao L.Y. (2020). Status and progress of kansei engineering design method. J. Mach. Des..

[34] Choi J. (2019). Material selection: Exploring the reliability of material perception data and its relevance to materials. Met. Mater. Int..

[35] Wan Q., Li X., Zhang Y., Song S., Ke Q. (2021). Visual perception of different wood surfaces: An event-related potentials study. Ann. For. Sci..

[36] Liang S., Pan C.R., Cen X.Q., Lin H., Li Q. (2022). Assessment of the Color Difference and Decay Resistance Performance of Moso Bamboo and Carbonized Moso Bamboo Treatments with Different Painting. For. Eng..

